# Cognitive Impairment in Cerebral Amyloid Angiopathy: A Single-Center Prospective Cohort Study

**DOI:** 10.3390/jcm13237427

**Published:** 2024-12-06

**Authors:** Aikaterini Theodorou, Athanasia Athanasaki, Konstantinos Melanis, Ioanna Pachi, Angeliki Sterpi, Eleftheria Koropouli, Eleni Bakola, Maria Chondrogianni, Maria-Ioanna Stefanou, Efthimios Vasilopoulos, Anastasios Kouzoupis, Georgios P. Paraskevas, Georgios Tsivgoulis, Elias Tzavellas

**Affiliations:** 1 Second Department of Neurology, “Attikon” University Hospital, School of Medicine, National and Kapodistrian University of Athens, 12462 Athens, Greece; katetheo24@gmail.com (A.T.); athanasia.athan@yahoo.gr (A.A.); melaniskos@gmail.com (K.M.); pachiioanna@gmail.com (I.P.); angste1995@gmail.com (A.S.); koropouli_ria@yahoo.gr (E.K.); elbakola@yahoo.gr (E.B.); mariachondrogianni@hotmail.gr (M.C.); marianna421@hotmail.co.uk (M.-I.S.); geoprskvs44@gmail.com (G.P.P.); 2 Department of Neurology & Stroke, Eberhard-Karls University of Tübingen, 72076 Tübingen, Germany; 3 Hertie Institute for Clinical Brain Research, Eberhard-Karls University of Tübingen, 72076 Tübingen, Germany; 4 First Department of Psychiatry, “Aiginition” Hospital, School of Medicine, National and Kapodistrian University of Athens, 11528 Athens, Greece; efvasilop@med.uoa.gr (E.V.); akouzoup@med.uoa.gr (A.K.); etzavell@med.uoa.gr (E.T.); 5 Department of Neurology, University of Tennessee Health Science Center, Memphis, TN 38163, USA

**Keywords:** cerebral amyloid angiopathy, cognitive impairment, neurocognitive testing, assessment, neuroimaging markers

## Abstract

**Background/Objectives:** Cognitive impairment represents a core and prodromal clinical feature of cerebral amyloid angiopathy (CAA). We sought to assess specific cognitive domains which are mainly affected among patients with CAA and to investigate probable associations with neuroimaging markers and Cerebrospinal Fluid (CSF) biomarkers. **Methods:** Thirty-five patients fulfilling the Boston Criteria v1.5 or v2.0 for the diagnosis of probable/possible CAA were enrolled in this prospective cohort study. Brain Magnetic Resonance Imaging and CSF biomarker data were collected. Every eligible participant underwent a comprehensive neurocognitive assessment. Spearman’s rank correlation tests were used to identify possible relationships between the Addenbrooke’s Cognitive Examination—Revised (ACE-R) sub-scores and other neurocognitive test scores and the CSF biomarker and neuroimaging parameters among CAA patients. Moreover, linear regression analyses were used to investigate the effects of CSF biomarkers on the ACE-R total score and Mini-Mental State Examination (MMSE) score, based on the outcomes of univariate analyses. **Results:** Cognitive impairment was detected in 80% of patients, and 60% had a coexistent Alzheimer’s disease (AD) pathology based on CSF biomarker profiles. Notable correlations were identified between increased levels of total tau (t-tau) and phosphorylated tau (p-tau) and diminished performance in terms of overall cognitive function, especially memory. In contrast, neuroimaging indicators, including lobar cerebral microbleeds and superficial siderosis, had no significant associations with cognitive scores. Among the CAA patients, those without AD had superior neurocognitive test performance, with significant differences observed in their ACE-R total scores and memory sub-scores. **Conclusions:** The significance of tauopathy in cognitive impairment associated with CAA may be greater than previously imagined, underscoring the necessity for additional exploration of the non-hemorrhagic facets of the disease and new neuroimaging markers.

## 1. Introduction

Cerebral amyloid angiopathy (CAA) represents a distinct small vessel disease, mainly affecting the small cortical and leptomeningeal cerebral arterioles [1]. CAA is considered a disease of the elderly, with an estimated prevalence of approximately 50–60% among demented elderly populations and a high coexistence rate with Alzheimer’s disease [1,2]. The diagnosis of probable or possible CAA in living patients without conducting a brain biopsy is based on the modified Boston criteria or the most recently published Boston Criteria version 2.0 for sporadic CAA, whereas histopathological evidence from brain autopsy or biopsy is demanded for a definite diagnosis [3,4].

Focal neurological deficits due to spontaneous lobar intracerebral hemorrhage (ICH), transient focal neurological episodes (TFNEs), and cognitive impairment or dementia are the main clinical presentations of CAA [4,5,6]. The characteristic neuroimaging findings of the disease include lobar ICH, convexity subarachnoid hemorrhage (cSAH), cortical superficial siderosis (cSS), cerebral microbleeds (CMBs) with a lobar distribution, enlarged perivascular spaces (EPVSs) in the centrum semiovale (CSO), and white matter hyperintensities [4,5,6].

The subclinical underlying pathology of the disease seems to begin approximately 30 years before the manifestation of the hemorrhagic lesions, and cognitive impairment has been suggested to represent a prodromal clinical manifestation even in the absence of the characteristic lobar ICH [7]. Nevertheless, the aspects of cognitive impairments implicated in the disease remain unclear [8]. In the literature, it has been argued that among patients with CAA, similar to those with vascular dementia, processing speed and executive functions are the most affected domains [9,10,11,12]. Memory seems to be less impaired [13]. Moreover, due to the predilection of the CAA pathology for the posterior lobes, impairment of visuo-perceptual processing has been also suggested [14].

In this prospective, single-center cohort study, we primarily sought to investigate the cognitive aspects among patients diagnosed with CAA. Additionally, we aimed to assess possible correlations among different Cerebrospinal Fluid (CSF) biomarkers and neuroimaging markers and the cognitive features of CAA. Possible differences in the neurocognitive profile among CAA patients with or without a coexistent Alzheimer’s disease (AD) pathology were investigated.

## 2. Subjects and Methods

### 2.1. Methods and Data Availability Statement

The datasets used and analyzed during the current study are included in this article. More detailed datasets are available from the corresponding author on reasonable request. This study was performed in accordance with the Strengthening the Reporting of Observational Studies in Epidemiology (STROBE) guidelines for reporting observational research [15]. Written informed consent was obtained from all patients or their legal representatives before enrollment.

### 2.2. Participants

Participants were recruited from a prospective cohort of patients diagnosed with CAA in our stroke service or from our outpatient stroke and dementia clinic at the Second Department of Neurology at “ATTIKON” University Hospital during a six-year period (2018–2024). This study was in accordance with the Declaration of Helsinki principles, and institutional review board approval was obtained from the Ethics Committee of “Attikon” University Hospital (decision number: EBD 499/8-9-2020).

Participants were included if (a) they were diagnosed with possible or probable CAA according to the modified Boston criteria or the more recently published Boston Criteria v2.0 for CAA [3,4], (b) they completed a comprehensive neuropsychological assessment, (c) they underwent brain Magnetic Resonance Imaging (MRI) within 28 days of the neuropsychological assessment, and (d) they underwent lumbar puncture within 28 days of the neuropsychological assessment. The CSF Aβ40, Aβ42, tau phosphorylated at threonine 181 (p-tau), and total tau levels were quantified. A lumbar puncture was performed either at the time of diagnosis/first presentation–assessment or at least 3 months after ICH onset in case of patients with acute ICH. Coexistent AD was diagnosed based on the A/T/N classification [16].

Patients were excluded from this cohort study if (a) they were diagnosed with CAA-related inflammation or with hereditary or iatrogenic CAA; (b) they had neuroimaging findings that were not related to an underlying CAA-related pathology/cause or were indicative of another neurological disorder; (c) they had any indication of alternative diagnoses, including signs of parkinsonism, signs of normal-pressure hydrocephalus, etc.; (d) they had either genetic CAA or any indication of genetic/familial AD; or (e) they did not provide written informed consent to participate in the current study. Moreover, patients with neurological symptoms including aphasia and patients who were unable to cooperate were also excluded.

### 2.3. MRI Acquisition

All brain MRI results were evaluated by two independent investigators (A.T. and G.T.) who were blinded to clinical characteristics. All patients underwent either a 1.5 Tesla (Philips Healthcare, Best, The Netherlands) or, preferably, a 3 Tesla MRI scan (Siemens Magnetom Prisma, Siemens Healthineers, Erlangen, Germany). A comprehensive brain MRI protocol was used in all participants, which included the following sequences: the gradient recalled echo (GRE) T2*-weighted imaging sequence or, preferably, the Susceptibility-Weighted Imaging (SWI) sequence, the T2-weighted sequence, the 3D fluid-attenuated inversion recovery (FLAIR) sequence, the diffusion-weighted imaging (DWI) sequence, and the post-contrast T1-weighted sequence.

Characteristic neuroimaging markers were assessed and documented. The presence and number of CMBs were evaluated on GRE-T2* or SWI [17]. The presence of cSS, focal (restricted to ≤3 sulci) or disseminated (>3 sulci), was also assessed [18]. Moreover, cSAH was detected in blood-sensitive MRI sequences with characteristic blooming limited to the subarachnoid space of the convexities [19]. Non-hemorrhagic markers were also detected, including EPVSs in CSO and the characteristic pattern of white matter hyperintensities [20,21].

### 2.4. Cerebrospinal Fluid Biomarkers

Lumbar puncture was performed in all eligible patients in accordance with our department’s protocol. The CSF was collected in polypropylene tubes, centrifuged, aliquoted, and stored in polypropylene tubes at −80 °C, whereas samples with more than 500 red blood cells/μL were discarded. The CSF Aβ40, Aβ42, tau, and p-tau levels were blindly quantified in duplicate using the Lumipulse chemiluminescent immunoassay (Fujirebio, Gent, Belgium). The samples were analyzed in different batches; however, we adhered to strict standard operating procedures and the manufacturer’s instructions.

### 2.5. Assessment of Cognitive Function

Following a complete physical and neurological examination, a neurocognitive evaluation of the global cognitive function and multiple specific domains using different tests was performed. Neuropsychological assessments were conducted once, during the initial evaluation of the patients or 3 months following an intracerebral hemorrhage, and always close to the lumbar puncture. However, the diagnosis of possible or probable CAA was based not only on the neuropsychological assessments but on the clinical phenotype, on the CSF biomarker profile, and on neuroimaging findings as well. More specifically, we sought to cross-check the medical history and the manifestations regarding memory decline with the patients’ caregivers. The detailed neurocognitive assessment included the use of the Mini-Mental State Examination (MMSE), the Frontal Assessment Battery (FAB), the Executive Clock Drawing Task (CLOX1 and CLOX2), and the Addenbrooke’s Cognitive Examination—Revised (ACE-R). Performance on these tests was scored according to published standardized normative data [22,23,24,25]. Patients with an ACE-R score of <88 were characterized as cognitive-impaired [25].

Patients with neurological symptoms including aphasia and patients who were unable to cooperate were excluded.

## 3. Statistical Analysis

If normally distributed, continuous variables were expressed as the mean ± standard deviation. Otherwise, they were reported as medians with interquartile ranges (IQRs). Continuous variables were tested with Student’s *t*-test (if normally distributed) or the Mann–Whitney U-test (if non-normally distributed). The chi-square test (sample size > 5) or the Fisher exact test (sample size ≤ 5) was used for dichotomized variables.

Correlations between continuous variables were assessed using Spearman’s rank correlation test. In addition, we used linear regression models to evaluate the effects of CSF biomarkers on the ACE-R total score and MMSE score, based on the outcomes of the univariate analyses. Multivariate logistic regression models were also used so that possible independent predictors for the total ACE-R score and MMSE score could be found, after adjusting for age, sex, years of education, and arterial hypertension. Multicollinearity was identified and assessed using the Variance Inflation Factor (VIF). Moreover, we used radar plots to visualize differences in multiple cognitive domains (total ACE-R and sub-scores) among CAA patients with and without AD coexistence. Differences were considered statistically significant at a value of *p* < 0.05. All statistical analyses were conducted using R software version 2023.06.0+421 [26].

## 4. Results

### 4.1. Participant Characteristics

This cohort consisted of 35 patients who fulfilled the criteria for the diagnosis of possible/probable CAA and completed the comprehensive neurocognitive assessment. The baseline characteristics of this cohort, including demographics, clinical features, neuroimaging findings, CSF biomarker levels, and the results of the neurocognitive assessments, are summarized in Table 1. The mean age of the study population was 70 ± 8 years, and 43% (15/35) were men. No statistically significant differences were detected among males and females with regard to different neurocognitive assessments (Table 2).

### 4.2. Clinical Features, Neuroimaging Markers, and Cerebrospinal Fluid Biomarker Levels

Among the study participants, the prevalence rates of focal neurological deficits, TFNEs, and cognitive impairment were 60% (21/35), 23% (8/35), and 80% (28/35), respectively. In all, 20% (7/35) of the patients complained of headache at the initial presentation, whereas 9% (3/35) and 43% (15/35) presented with seizures and behavioral changes/psychiatric manifestations, respectively.

Ninety-four percent (33/35) of the participants had lobar CMBs, whereas twenty-nine percent (10/35) presented with lobar ICH. cSS and cSAH were present in 46% (16/35) and 9% (3/35) of patients, respectively. Non-hemorrhagic lesions including EPVSs in CSO, the multispot hyperintense white matter pattern, and cortical microinfarcts were detected in 44% (15/35), 53% (18/35), and 17% (6/25) of participants, respectively.

Lumbar puncture was performed in all participants included in this study. The results demonstrated mean Aβ40 levels of 7655 ± 2703 pg/mL, decreased mean Aβ42 levels of 421 ± 179 pg/mL (cut-off value for CSF Aβ42: 520 pg/mL), increased mean t-tau levels of 522 ± 327 pg/mL (cut-off value for CSF total tau: 360 pg/mL), and increased mean p-tau levels of 71 ± 38 pg/mL (cut-off value for CSF p-tau: 60 pg/mL). The Aβ42/Aβ40 ratio was 0.076 ± 0.038 (cut-off value for Aβ42/Aβ40 ratio: 0.063).

Based on the A/T/N classification scheme for AD biomarkers, 60% (21/35) of the study participants were classified as having coexistent AD [16].

### 4.3. Associations of ACE-R Sub-Scores and Other Neurocognitive Tests with CSF and Neuroimaging Parameters Among Patients with CAA

**Figure 1:**
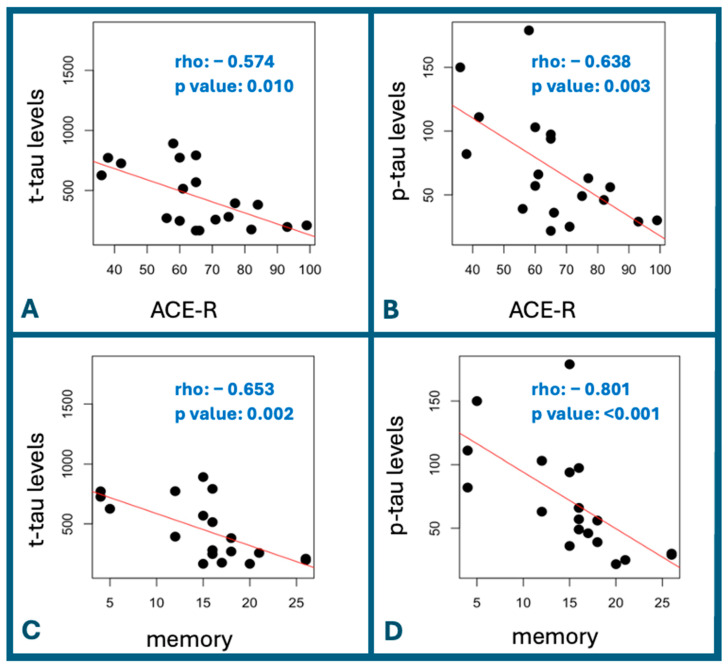
Correlations between the levels of CSF t-tau and p-tau and the total ACE-R scores and memory sub-scores among the CAA participants. Legend: Negative correlations are depicted between the CSF t-tau levels and ACE-R (**A**), p-tau levels and ACE-R (**B**), t-tau levels and memory sub-score (**C**), and p-tau levels and memory sub-score (**D**). The Spearman’s rank correlation test was used to determine significant correlations.

Table 3 and Table 4 and Figure 1 summarize the relationships between ACE-R sub-scores and other neurocognitive test results and the biomarker and neuroimaging parameters among CAA patients. Significant negative correlations were found between the total ACE-R score (Figure 1A,B) and memory sub-scores (Figure 1C,D) and the levels of t-tau and p-tau. P-tau levels were also marginally correlated with the language sub-score of ACE-R. T-tau and p-tau levels were also negatively correlated with the MMSE score, whereas p-tau levels were significantly negatively correlated with the CLOX2 test result. Neuroimaging markers were not significantly correlated with the different neurocognitive assessments.

### 4.4. Comparison of Neurocognitive Profiles Among CAA Patients with and Without Coexistent AD

**Figure 2:**
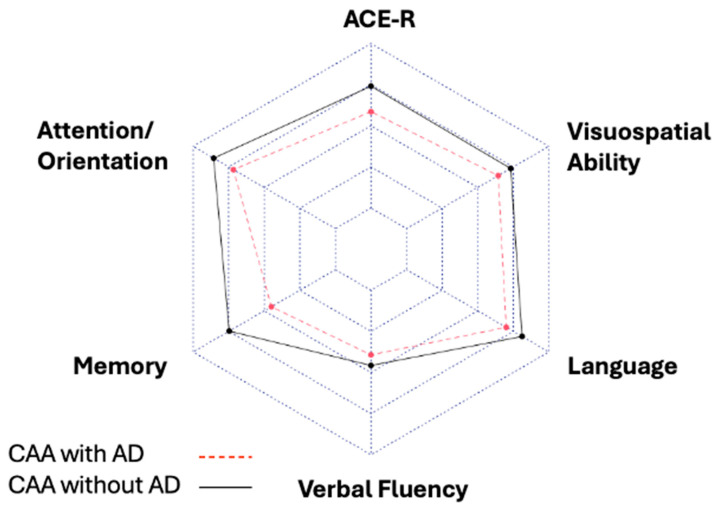
Radar plot depicting differences in multiple cognitive domains (total ACE-R and sub-scores) among CAA patients with and without AD coexistence. Legend: Statistically significant differences were detected in total ACE-R scores and memory sub-scores among CAA patients with and without coexistent AD.

Table 5 summarizes the multiple comparison results for the different neurocognitive tests among CAA patients with or without coexistent AD. Statistically significant differences were detected in the total ACE-R scores and memory sub-scores among these subgroups; these differences are illustrated in a characteristic radar plot (Figure 2). Despite the fact that CAA patients without AD coexistence had better scores in all the neurocognitive tests, no other significant differences were disclosed.

### 4.5. Independent Predictors of Impaired ACE-R and MMSE Scores

Multivariate logistic regression models were computed including the total ACE-R score and MMSE score as independent variables and adjusting for age, years of education, sex, and arterial hypertension (Table 6). Mainly low multicollinearity was observed, without requiring further investigation. Increased p-tau levels remained the only factor independently associated with reduced ACE-R (β: −0.309, *p*: 0.032) and MMSE scores (β: −0.066, *p*: 0.052) in CAA patients.

## 5. Discussion

The primary objective of our research was to thoroughly examine the correlations among clinical characteristics, neuroimaging markers, CSF biomarker levels, and cognitive decline in patients with CAA. Our study revealed a prevalence of cognitive impairment of 80%, with 60% (21/35) of subjects diagnosed with concurrent AD. This finding corresponds with those of earlier studies, which indicated that the prevalence of dementia or mild cognitive impairment (MCI) in individuals with CAA varies between 50% and 98% [1,2,6,27,28]. The notable prevalence of concurrent AD pathology in individuals with CAA also aligns with earlier research results [28,29].

Neuroimaging markers exhibited no significant correlation with the different neurocognitive evaluations, in contrast to prior research findings. Cerebral microbleeds (CMBs) have been observed in roughly 40% of patients with AD [30] and have been associated with cognitive decline and increased mortality [31,32,33,34,35,36,37,38]. Cortical microinfarcts and overall brain atrophy have also been associated with deteriorating cognitive performance [11,39]. Post-ICH dementia has been associated with various neuroimaging markers, such as a heightened white matter hyperintensity (WMH) load, disseminated cSS, and an enhanced small vascular disease (SVD) score [10]. Individuals with cSS have exhibited AD-related characteristics, including memory decline and hippocampal atrophy [29]. A meta-analysis also established a correlation between cSS and AD in patients with CAA [35]. The advancement of CAA from posterior to anterior lobe regions of the brain, especially in conjunction with a deterioration in executive function, has been identified as a significant marker of co-occurrence with AD [37]. The relatively small sample size and differences in methodologies may have contributed to discrepancies in our findings.

Patients with CAA and concomitant AD exhibited significantly worse ACE-R and memory scores than did those with CAA alone. To the best of our knowledge, this was the first time that the correlations of different clinical, neuroimaging, and biological markers with specific cognitive domains were researched, although there is scarce evidence for the effects of CAA on memory and executive function [29,37].

The total tau and p-tau levels were negatively correlated with ACE-R scores, memory sub-scores, and MMSE scores. This was also depicted in the multivariable logistic regression models, where increased p-tau levels were the sole independent variable correlated with diminished ACE-R and MMSE scores in CAA patients. These results may underscore a possible significant role for tau pathology in the pathogenesis of CAA and its co-existence with AD and as a probable novel biomarker in distinguishing CAA from other small vessel diseases.

Indeed, tau may assume a more significant role in cognitive deterioration associated with CAA than previously acknowledged [38,40,41], particularly in advanced disease stages or instances of CAA coexisting with AD [42]. Jack et al. elucidated that, whereas amyloid-beta triggers pathogenic alterations, tau is responsible for neuronal damage and cognitive deterioration [43]. Cohort studies indicate that tau in CAA may directly influence cognitive performance, possibly due to compromised perivascular clearance and neurovascular dysfunction [44,45]. Nonetheless, discerning whether cognitive impairment stems from AD, CAA, or a combination of both still presents a significant challenge.

CAA and AD are regarded as disorders with intersecting pathogenic processes. Vascular impairment and neurodegenerative processes may interact in both conditions, with vascular disease acting as a main contributor to brain structural loss, rather than merely a subsequent consequence of neurodegeneration [46]. CAA has been demonstrated to significantly contribute to cortical atrophy [47], perhaps serving as a common endpoint between CAA and AD. Both disorders are distinguished by the formation of amyloid-beta (Aβ), which is a fundamental aspect of their pathology [27,48]. Patients with CAA are believed to have compromised Aβ clearance via the glymphatic system and intramural perivascular drainage. The existence of perivascular tau neurites encircling amyloid-laden arteries may indicate a pathogenic intersection between the two conditions [41].

However, both conditions may independently induce brain damage while potentially aggravating each other in coexistence. Aβ-induced neuronal death and subsequent tau tangle development seem pivotal to the neurodegeneration seen in AD [49]. Even so, amyloid build-up cannot be easily ascribed to a specific pathological process [1]. Tau deposition in CAA may have a specific or varied pattern [50]. Interestingly, research on hereditary CAA, characterized by reduced AD pathology, has suggested that hippocampal atrophy is not inherently associated with vascular Aβ accumulation, akin to the build-up of hyperphosphorylated tau in advanced sporadic CAA [46].

Finally, in light of the biological definitions and molecular tracing through the distinct biomarker profiles of many neurodegenerative entities, it is crucial to align the CAA research framework accordingly [13,51,52]. A biological-based approach to CAA would, firstly, enhance the sensitivity and specificity of the diagnostic criteria for CAA in the future, especially for possible CAA [53]. Τhe integration of a potential indicative CSF biomarker profile could also serve as an ideal substrate for the development of tau-targeted treatments, pioneering a challenging era of therapeutics in CAA. Furthermore, this pattern would facilitate the development of an effective staging system, providing a critical tool for assessing disease severity. Novel blood biomarkers such as plasma phosphorylated tau isoforms (pTau181, 217, and 231) will be of great importance, taking into account the cost, the convenience, and the high diagnostic accuracy already proven in AD [54,55].

### Limitations

Notwithstanding the comprehensive investigation of clinical characteristics, neuroimaging markers, and CSF biomarker levels related to cognitive deterioration in CAA patients, our study has specific limitations. The limited sample size (*n* = 35) constrains the capacity to identify nuanced, clinically significant associations. This is due to the limited generalizability of our results, the lack of precision and reliability, and the limited ability for heterogeneity exploration. Therefore, taking into account the small sample size in combination with the single-center design, the results of the present study should be presented with caution in terms of their generalizability. The utilization of both 1.5 T and 3 T MRI scanners may have resulted in variability in the identification of smaller hemorrhagic lesions, such as CMBs and cSS, and non-hemorrhagic lesions including white matter FLAIR hyperintensities, cortical microinfarcts, and leptomeningeal enhancement [16]. This limitation, in association with the small sample size, could also explain the non-significant correlations between neuroimaging markers and different neurocognitive evaluations. At the same time, the absence of distinct MRI indicators for tau pathology represents a significant deficiency in neuroimaging [43], highlighting, at the same time, the necessity for molecular imaging biomarkers to directly detect cerebrovascular amyloid in vivo [49]. The lack of volumetric analyses of brain MRI of the subjects, specifically a hippocampal volume assessment, or a CAA score, that could elucidate the relationship between CAA, MCI, and dementia [42,56], constitutes another limitation, particularly as research increasingly advocates for multimodal strategies to enhance diagnostic accuracy in CAA progression [37,56]. Lastly, the cross-sectional design of our study is another drawback, underscoring the necessity for longitudinal, multi-center cohort studies to ascertain whether tau accumulation safely predicts cognitive impairment in CAA patients.

## 6. Conclusions

Our findings underscore the pivotal role of tau pathology in cognitive deterioration in CAA, irrespective of amyloid buildup or conventional neuroimaging markers. This highlights the significance of integrating tau biomarkers into clinical assessments of CAA for diagnostic and prognostic objectives. Tau biomarkers could also contribute to the discrimination of CAA patients with and without coexistent AD. The advancement of direct molecular imaging biomarkers is an unmet requirement that could greatly enhance early diagnosis, risk stratification, and the formulation of disease-modifying therapeutics for both CAA and AD, as well as their intersection. Future research should prioritize monitoring the progression of tau pathology and cognitive deterioration over time in patients with CAA. Such evidence may provide novel strategies for mitigating cognitive deficits in persons with CAA, especially those with concurrent AD.

## Figures and Tables

**Table 1:** Baseline characteristics of our study population (*n* = 35).

Variable	Overall (N = 35)
Age at presentation (years), mean (SD)	70 (7.9)
Sex—Male, *n* (%)	15 (42.9)
History of brain trauma, *n* (%)	0 (0.0)
Vascular risk factors	
Arterial hypertension, *n* (%)	21 (60.0)
Diabetes Mellitus II, *n* (%)	8 (22.9)
Hyperlipidemia, *n* (%)	20 (57.1)
Clinical signs at presentation	
Signs of intracerebral hemorrhage, *n* (%)	9 (25.7)
Focal neurological signs, *n* (%)	21 (60)
TFNEs, *n* (%)	8 (22.9)
Cognitive impairment, *n* (%)	28 (80)
Headache, *n* (%)	7 (20)
Seizures, *n* (%)	3 (8.6)
Behavioral changes/psychiatric signs, *n* (%)	15 (42.9)
Neuroimaging findings at presentation	
SWI vs. GRE/T2*, *n* (%)	26 (74.3)
Lobar hemorrhage, *n* (%)	10 (28.6)
Lobar cerebral microbleeds, *n* (%)	33 (94.3)
Cerebellar microbleeds, *n* (%)	15 (42.9)
cSAH, *n* (%)	3 (8.6)
cSS, *n* (%)	16 (45.7)
Disseminated cSS vs. focal cSS, *n* (%)	10 (62.5)
Cerebellar cSS, *n* (%)	0 (0.0)
Enlarged perivascular spaces in centrum semiovale, *n* (%)	15 (44.1)
Multispot WMH pattern, *n* (%)	18 (52.9)
Cortical microinfarcts, *n* (%)	6 (17.1)
Gd+ enhancement, *n* (%)	0 (0.0)
CSF biomarkers and APOE genotype	
Amyloid Aβ40, mean (SD)	7655 (2702.7)
Amyloid Aβ42, mean (SD)	421.2 (179.1)
total tau (t-tau), mean (SD)	522.3 (326.9)
phosphorylated tau (p-tau), mean (SD)	70.89 (37.7)
Aβ42/Aβ40, mean (SD)	0.062 (0.035)
AD coexistence, *n* (%)	21 (60)
Neurocognitive assessment	
Years of education, mean (SD)	8.6 (4.7)
ACE-R (total score), mean (SD)	65.9 (16.9)
ACE-R (attention), mean (SD)	14.1 (3.9)
ACE-R (memory), mean (SD)	15.4 (6.2)
ACE-R (fluency), mean (SD)	5.9 (3.3)
ACE-R (language), mean (SD)	19.6 (4.6)
ACE-R (visuospatial), mean (SD)	10.9 (2.8)
MMSΕ, mean (SD)	21.8 (5.1)
FAB, mean (SD)	11.5 (3.8)
CLOX1, mean (SD)	7.4 (4.1)
CLOX2, mean (SD)	10.1 (3.3)

SD: standard deviation.

**Table 2:** Comparison of different neurocognitive tests among males and females with CAA.

Characteristics	MALES	FEMALES	*p*-Value
ACE-R	63.8	67.9	0.610
Attention/orientation	12.8	15.3	0.162
Memory	15.3	15.4	0.982
Fluency	5.1	6.7	0.301
Language	19.4	19.7	0.908
Visuospatial	11.1	10.8	0.817
MMSE	21.4	22.1	0.713
CLOX1	7.0	7.7	0.709
CLOX2	9.2	10.8	0.284
FAB	10.1	12.4	0.174

**Table 3:** The correlations between sub-scores of ACE-R and CSF biomarkers and neuroimaging findings.

	ACE-R		Attention/Orientation		Memory		Fluency		Language		Visuospatial	
	Rho	*p* Value	Rho	*p* Value	Rho	*p* Value	Rho	*p* Value	Rho	*p* Value	Rho	*p* Value
Age at examination	−0.156	0.524	0.026	0.916	−0.318	0.185	−0.158	0.519	−0.105	0.668	−0.111	0.651
Sex	0.116	0.637	0.273	0.257	−0.058	0.813	0.243	0.315	−0.039	0.875	−0.097	0.693
Aβ40	0.163	0.546	0.233	0.384	−0.340	0.197	0.275	0.303	0.343	0.194	0.167	0.536
Aβ42	0.215	0.378	−0.062	0.799	0.351	0.141	−0.054	0.826	0.137	0.577	0.099	0.684
t-tau	−0.574	0.010	−0.375	0.114	−0.653	0.002	−0.298	0.215	−0.382	0.107	−0.317	0.186
p-tau	−0.638	0.003	−0.410	0.081	−0.801	<0.001	−0.286	0.236	−0.457	0.049	−0.446	0.055
Aβ42/Aβ40	0.014	0.959	−0.262	0.345	0.385	0.157	−0.141	0.616	−0.217	0.436	−0.049	0.864
Lobar hemorrhage	0.010	0.966	0.0001	0.999	−0.021	0.932	−0.188	0.440	−0.176	0.470	−0.198	0.417
Multiple lobar CMBs (>10)	0.145	0.553	0.053	0.828	0.213	0.382	0.107	0.664	0.106	0.666	−0.039	0.871
Cerebellar microbleeds	−0.203	0.406	−0.078	0.751	0.009	0.969	−0.185	0.448	−0.116	0.637	−0.271	0.261
cSS	−0.155	0.525	−0.021	0.932	−0.396	0.093	0.073	0.766	−0.291	0.228	−0.354	0.137
Enlarged perivascular spaces in centrum semiovale	0.068	0.784	0.137	0.577	−0.223	0.359	0.0001	0.999	−0.087	0.723	0.029	0.906
Multispot WMH pattern	0.164	0.502	0.195	0.423	−0.068	0.782	0.146	0.551	−0.019	0.937	0.145	0.553
Cortical microinfarcts	−0.109	0.654	−0.222	0.360	−0.158	0.519	−0.048	0.847	0.079	0.749	0.0001	0.999

**Table 4:** The correlations between different neurocognitive tests and CSF biomarkers and neuroimaging findings.

	MMSE		CLOX1		CLOX2		FAB	
	Rho	*p* Value	Rho	*p* Value	Rho	*p* Value	Rho	*p* Value
Age at examination	−0.071	0.736	−0.257	0.248	−0.533	0.011	−0.246	0.271
Sex	0.084	0.689	0.081	0.720	0.118	0.600	0.307	0.164
Aβ40	−0.074	0.778	0.009	0.976	0.067	0.820	0.013	0.964
Aβ42	0.043	0.838	0.206	0.357	0.394	0.069	0.150	0.504
t-tau	−0.415	0.039	−0.223	0.319	−0.468	0.028	−0.340	0.121
p-tau	−0.451	0.024	−0.344	0.117	−0.600	0.003	−0.358	0.102
Aβ42/Aβ40	−0.004	0.987	0.314	0.274	0.305	0.288	0.046	0.875
Lobar hemorrhage	0.185	0.376	−0.195	0.385	0.024	0.914	0.162	0.472
Multiple lobar CMBs (>10)	0.299	0.146	0.060	0.789	0.130	0.564	0.412	0.057
Cerebellar microbleeds	−0.089	0.672	−0.268	0.228	−0.254	0.253	−0.014	0.949
cSS	−0.191	0.359	−0.073	0.746	−0.220	0.324	−0.364	0.096
Enlarged perivascular spaces in centrum semiovale	0.028	0.894	−0.045	0.842	−0.113	0.615	−0.157	0.485
Multispot WMH pattern	0.150	0.473	−0.029	0.898	−0.139	0.538	0.087	0.701
Cortical microinfarcts	−0.357	0.079	−0.232	0.299	−0.212	0.344	−0.378	0.083

**Table 5:** Comparison of different neurocognitive tests among CAA patients with and without coexistent AD.

Characteristics	CAA Without AD	CAA with AD	*p*-Value
ACE-R	74.1	58.6	0.042
Attention/orientation	15.4	12.9	0.158
Memory	19.4	11.7	0.003
Fluency	6.4	5.5	0.543
Language	21.1	18.2	0.175
Visuospatial	11.7	10.3	0.301
MMSE	23.5	20.4	0.132
CLOX1	7.8	7.2	0.736
CLOX2	10.9	9.6	0.379
FAB	12.0	11.1	0.588

**Table 6:** The results of the linear regression models for ACE-R and MMSE based on CSF biomarkers and possible confounders among CAA patients.

Covariate/Factor	Estimate	*p* Value	Lower 95%	Upper 95%	Adjusted Estimate *	VIF **	*p* Value
ACE-R							
Aβ40	0.001	0.894	−0.003	0.004	0.002	2.478	0.761
Aβ42	0.013	0.518	−0.029	0.055	0.006	2.112	0.860
t-tau	−0.041	0.005	−0.068	−0.014	−0.032	2.614	0.317
p-tau	−0.235	0.006	−0.395	−0.076	−0.347	5.412	0.038
Aβ42/Aβ40	29.130	0.829	−255.672	313.932	−219.979	2.756	0.610
Age	−0.439	0.371	−1.448	0.569			
Years of education	2.189	0.032	0.230	4.149			
Sex	4.122	0.610	−12.616	20.860			
Arterial hypertension	−2.345	0.780	−19.767	15.076			
MMSE							
Aβ40	−0.0002	0.487	−0.001	0.001	−0.0002	2.436	0.864
Aβ42	0.005	0.410	−0.007	0.017	0.002	2.078	0.807
t-tau	−0.011	0.008	−0.019	−0.003	−0.007	2.463	0.195
p-tau	−0.061	0.013	−0.107	−0.014	−0.070	3.233	0.039
Aβ42/Aβ40	13.560	0.707	−62.264	89.394	−20.201	2.458	0.819
Age	−0.085	0.549	−0.373	0.204			
Years of education	0.527	0.047	0.008	1.047			
Sex	0.779	0.713	−3.545	5.108			
Arterial hypertension	2.890	0.164	−1.269	7.048			

* Adjustment for age, years of education, sex, and arterial hypertension. ** VIF: Variance Inflation Factor.

## Data Availability

All data needed to evaluate the conclusions in the paper are present in the main manuscript. Additional data related to this paper may be requested from the corresponding author, upon reasonable request.
